# Supplementary material for: Identification of photoperiod-induced specific miRNAs in the adrenal glands of Sunite sheep (Ovis aries)
Source: Front Vet Sci. 2022 Jul 22;9:888207. doi: 10.3389/fvets.2022.888207 (PMC9354845; doi:10.3389/fvets.2022.888207)
Supplement: Supplementary file 1 [file Data_Sheet_1.ZIP › Supplementary file/miRDeep2 file.zip!/miRDeep2 file/Know_Structure/LP42A2/oar-mir-30c.pdf]

miRBase precursor : oar-mir-30c
Total read count : 46401
oar-miR-30c read count : 46239
remaining reads : 162

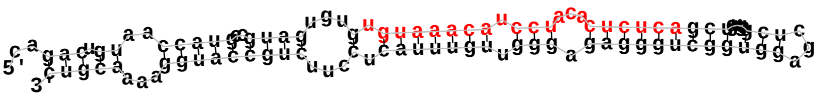

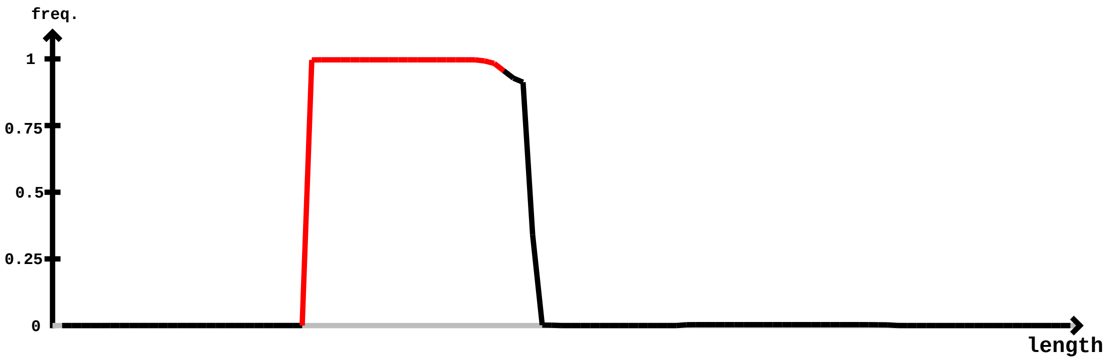

oar-miR-30c

```
5'- cagacuguaaccaugccguagugugUGUAAACAUCCUACACUCUCAgcugugagcucgagguggcugggagaggguuguuuacuccuucugccauggaaaacguc   -3'    exp
    ..(((.((..(((((..((((...(.(((((((.(((...(((((((((....(((...)))))))))))).))).))))))).)...)))))))))...)))))    reads    mm    sample
    cagacuguaaccaugccguagugug................................................................................    2    0    seq
    ....cuguaaccaugccguagugu.................................................................................    1    0    seq
    ....cuguaaccaugccguagugug................................................................................    1    0    seq
    .........................uguaaacauccuacacu...............................................................    4    0    seq
    .........................uguaaacauccCacacuc..............................................................    2    1    seq
    .........................uguaaacauccuacacuc..............................................................    133    0    seq
    .........................uguaaacauccuacacuU..............................................................    61    1    seq
    .........................uguGaacauccuacacuc..............................................................    1    1    seq
    .........................uguaaacauccuacacucu.............................................................    386    0    seq
    .........................uguaaacaCccuacacucu.............................................................    28    1    seq
    .........................uguGaacauccuacacucu.............................................................    2    1    seq
    .........................uguaaacauccuacacAcu.............................................................    1    1    seq
    .........................uguaaacauccuacacuUu.............................................................    1    1    seq
    .........................uguaaacauccuacacucuc............................................................    1212    0    seq
    .........................uguaaacaCccuacacucuc............................................................    78    1    seq
    .........................uguaaacauccuacacucuca...........................................................    1193    0    seq
    .........................uguaaacaCccuacacucuca...........................................................    90    1    seq
    .........................uguaaacauccuacacucucag..........................................................    632    0    seq
    .........................uguaaacaCccuacacucucag..........................................................    71    1    seq
    .........................uguaaacaCccuacacucucagc.........................................................    1805    1    seq
    .........................uguaaacauccuacacucucagc.........................................................    24730    0    seq
    .........................uguaaacaCccuacacucucagcu........................................................    1136    1    seq
    .........................uguaaacauccuacacucucagcu........................................................    14565    0    seq
    .........................uguaaacaCccuacacucucagcug.......................................................    2    1    seq
    .........................uguaaacauccuacacucucagcug.......................................................    23    0    seq
    .........................uguaaacaCccuacacucucagcugu......................................................    1    1    seq
    .........................uguaaacauccuacacucucagcugu......................................................    61    0    seq
    ..........................guaaacauccuacacucu.............................................................    1    0    seq
    ..........................guaaacauccuacacucuc............................................................    1    0    seq
    ..........................guaaacaCccuacacucuca...........................................................    1    1    seq
    ..........................guaaacauccuacacucucagc.........................................................    9    0    seq
    ..........................guaaacaCccuacacucucagc.........................................................    1    1    seq
    ..........................guaaacauccuacacucucagcu........................................................    7    0    seq
    ...........................uaaacauccuacacucucagc.........................................................    1    0    seq
    ................................................................cugggagaggguuguuuac......................    3    0    seq
```

oar-miR-30c

```
cagacuguaaccaugccguagugugcuguaaacauccuacacucucagcugugagcucgagguggcugggagaggguuguuuacuccuucugccauggaaaacguc
................................................................cugggagaggguuguuuacu.....................   19   0   seq
................................................................cugggagaggguuguuuacuc....................   14   0   seq
................................................................cugggagaggguuguuuacucc...................   94   0   seq
................................................................cugggagaggguuguuuacuccu..................   6    0   seq
................................................................cugggagaggguuguuuacuccuu.................   1    0   seq
.................................................................ugggagaggguuguuuacu.....................   1    0   seq
.................................................................ugggagaggguuguuuacuc....................   4    0   seq
.................................................................ugggagaggguuguuuacucc...................   16   0   seq
```
